# The sustainability of stock price fluctuations: Explanation from a recursive dynamic model

**DOI:** 10.1371/journal.pone.0255081

**Published:** 2021-08-17

**Authors:** Jun Xie, Wenqian Xia, Bin Gao

**Affiliations:** 1 School of Economics, Guangxi University, Nanning, China; 2 School of Economics, Guangxi University for Nationalities, Nanning, China; BeiHang University School of Economics and Management, CHINA

## Abstract

The sustainability of stock price fluctuations indicated by many empirical studies hardly reconciles with the existing models in standard financial theories. This paper proposes a recursive dynamic asset pricing model based on the comprehensive impact of the sentiment investor, the information trader and the noise trader. The dynamic process of the asset price is characterized and a numerical simulation of the model is provided. The model captures the features of the actual stock price that are consistent with the empirical evidence on the sustainability of stock price fluctuations. It also offers a partial explanation for other financial anomalies, for example, asset price’s overreaction, asset bubble and the financial crisis. The major finding is that investor sentiment is the key factor to understand the sustainability of stock price fluctuations.

## 1. Introduction

**Fig 1:**
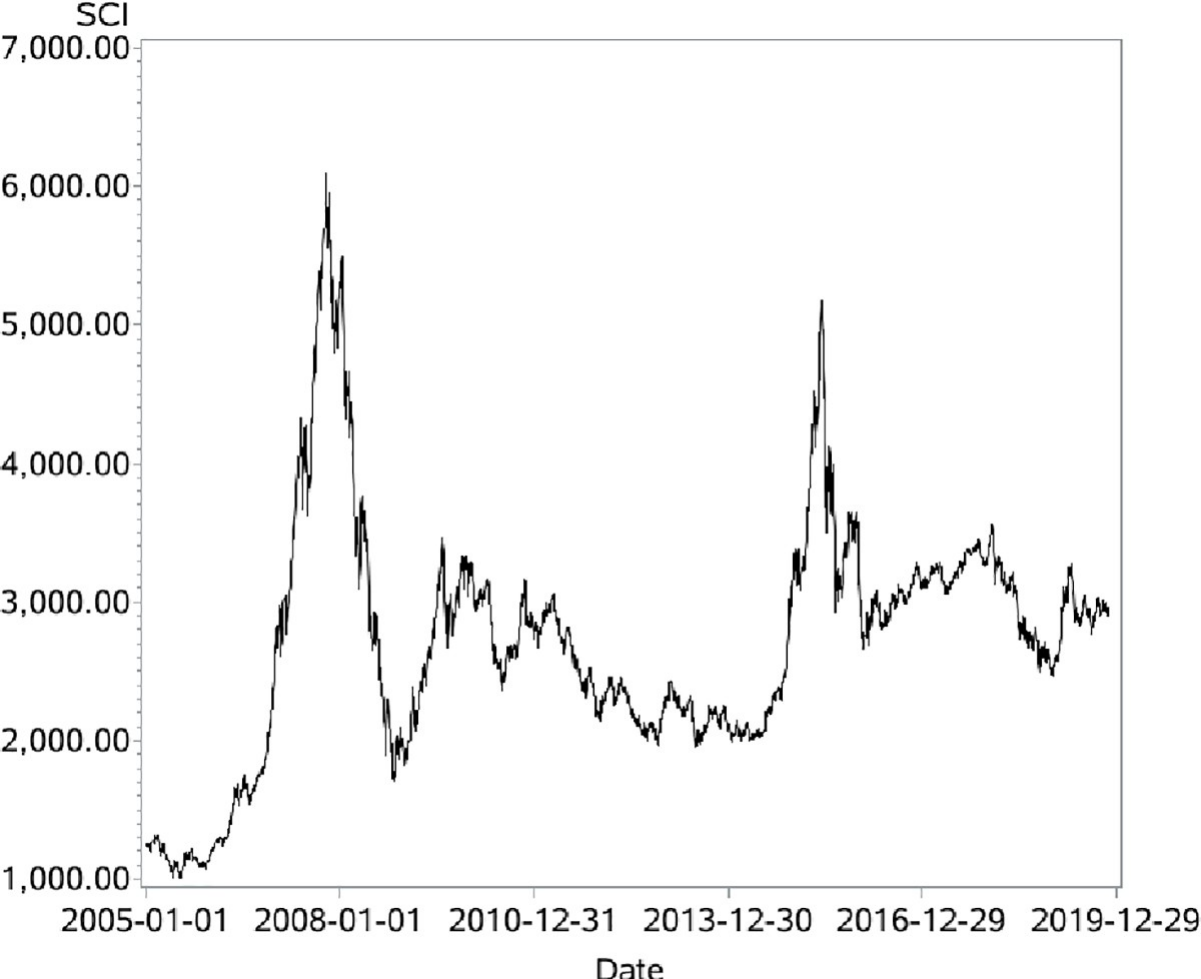
The closing price Shanghai Composite Index.

Stock price fluctuations are always higher than the expectation of the standard financial theory. In standard financial theories, such as CAPM [1], stock price changes only when new information appears, and volatility should be quite low. However, Schwert [2] uses monthly returns from 1802 to 2010, daily returns from 1885 to 2010, and intraday returns from 1982 to 2010 in the USA to show how stock price fluctuations have changed over time, and illustrate the historically high levels of stock price fluctuations in the USA. In fact, China has a similar situation. The Chinese stock market is a newly developing capital market where a dramatic fall in price always follows a soar and the price fluctuates repeatedly. For example, from June 6, 2005 to October 16, 2007, within two and a half years, Shanghai Composite Index (SCI) increased more than 6 times, rising from 998.23 points to 6124.04 points; from October 16, 2007 and August 11, 2008, within one year, SCI decreased more than half of the top points, dropping from 6124.04 points to 2470.07 (see Fig 1). The mean and standard deviation of SCI were 2720 and 868 respectively from 2005-01-01 to 2019-11-14. All these phenomena deviated from the Chinese stock market are unexplainable through standard financial theories.

The literature for possible clues of the sustainability of stock price fluctuations may go back to several seminal works. Abundant empirical evidences imply that stock price fluctuations are explained with the subsequent variation in the stock quality [3], economic fundamentals [4–6], overconfidence [7–9], investor attention [10], subjective beliefs [11], microeconomic foundation [12], industrial policy [13], corporate social responsibility [14], monetary policy [15] and sentiment [16]. The factors that affect the fluctuations of stock price may seem so numerous, but they can be roughly divided into two categories: (1) Fundamental factors (rational factors), which include the stock quality, economic fundamentals, industrial policy, monetary policy and so on. (2) Sentiment factors, which include overconfidence, investor attention, subjective beliefs, and so on, since overconfidence, investor attention and other subjective beliefs are always influenced by investor sentiment. In fact, lots of empirical literature [17–22] show that sentiment has a significant influence on the fluctuations of stock prices. In other words, the continuous fluctuation of a stock price is the result of the joint action of heterogeneous investors: rational investor and sentiment investor. The theoretical asset pricing model based on the heterogeneous is urgently needed to explain this phenomenon.

Asset pricing model based on the heterogeneous is a hot spot in the recent thirty years. DeLong, et al. [23] confirm that arbitrageurs buy in anticipation of positive-feedback trading by the Noise trader, and thus destabilize prices. Wang [24] presents a dynamic trading model with differentially informed investors and shows that less-informed investors can rationally behave like price chasers. Brock and Hommes [25] investigate the dynamics in a simple present discounted value asset pricing model with heterogeneous beliefs and show that price fluctuations are driven by the evolutionary dynamics between different expectation schemes (‘rational animal spirits’). Barlevy and Veronesi [26] consider a model with risk-neutral Outsiders trading with Noise trader and Insiders, optimally extracting information from the price of an asset. Mendel and Shleifer [27] present a model in which rational but uninformed traders occasionally chase noise as if it were information. In their model, noise trader can have an impact on market equilibrium disproportionate to their size in the market. Agliari, et al. [28] propose a stock market model in which participation depends upon an attractiveness measure related to the market activity and the fundamental value of the market. Oshima [29] builds a two-agent New Keynesian model with subjective and objective beliefs about capital gains from stock prices and shows that the presence of the two agents improves second moments of stock prices with realistic moments of business cycle properties. Schmitt, et al. [30] proposes a simple agent-based computational model in which speculators’ trading behavior may cause bubbles and crashes, excess volatility, serially uncorrelated returns, fat-tailed return distributions and volatility clustering. Few of them discuss the investor sentiment in their theoretical model based on the heterogeneous. In particular, there is no model based on the heterogeneous to describe the specific process of price changes under the influence of the sentiment investor. Li [31] examines the changes in price with the heterogeneous sentiment. But aforementioned empirical papers show that stock price fluctuations by the combined action of the rational investor (the information trader) and the sentiment investor, instead of heterogeneous sentiment investors. These phenomena indicate a gap between current theoretical models and empirical evidence.

To fill the gap between the asset pricing theory model and empirical model in the paper, a recursive dynamic asset pricing model with heterogeneous traders (the information trader, the sentiment trader and the noise trader) is proposed to explain in theory why the sustainability of stock price fluctuations appears in the real financial market. A similar model with heterogeneous agents is proposed by Hong and Stein [32]. This paper extends the analysis of the seminal work of Hong and Stein [32] in the following two aspects: (1) The effect of investor sentiment is included in our discussion whereas Hong and Stein [32] did not mention it. The paper analyzes investor sentiment instead of discussing the moment trader, since the seminal works [11, 17–22] confirm that investor sentiment is a key factor in asset pricing. For example, Adam et al. [11] suggest that asset price dynamics are to a large extent influenced by investors’ subjective optimism and pessimism. (2) This paper uses a discrete recursive method to build the model, yet Hong and Stein [32] use the continuous method. This makes the stock price simulated by the model in this paper more intuitive to show the sustainability of stock price fluctuations, and it is found that the sustainability of stock price fluctuations is closely related to sentiment investor which is consistent with the results of empirical literature [17–22].

Our paper contributes three fresh insights. Firstly, the paper derives a recursive dynamic asset pricing model with heterogeneous traders, which include the information trader, the sentiment trader and the noise trader. Secondly, the paper finds that investor sentiment is the key factor that leads the stock price to fluctuate powerfully and causes the sustainability of stock price fluctuations anomaly. Our third result explores the source of financial anomalies. From our model, we show that overreaction, asset bubble and the financial crisis are attributable to investor sentiment.

The rest of this paper is organized as follows. Section 2 presents the assumptions of the model; Section 3 describes the dynamic recursive asset pricing model with heterogeneous traders; Section 4 analyzes the model thoroughly and displays the dynamic process of stock price; Section 5 presents a numerical example and Section 6 is the conclusion.

## 2. The model assumptions

In the standard finance theories, all investors are rational and have homogeneous cognition. However, in the real financial market, the situation is different. As aforementioned empirical papers show that stock price fluctuations by the combined action of the rational investor (the information trader) and the sentiment investor, we suppose that there are three investors in the market: the information trader, the sentiment trader and the noise trader. The information trader trades based on the accounting information, which means the information trader is rational. The sentiment trader buys the asset when the sentiment runs high and sells the asset when sentiment falls. We simply assume that investor sentiment rises when asset prices rise and vice versa, and when the information trader (or the sentiment trader) enters or exits the market, the entering or exiting behavior equally affects the stock price in opposite directions. For simplicity, we only analyze the impact of positive information.

(i) The information trader

The information trader optimizes his or her investment by collecting the information himself, known as the private information. This type of trader does not care about the effect of the price change or the other investors’ behavior. In other words, the information trader is rational. Let *P*_*t*_ denote the stock price at the time *t*, and the stock price change is Δ*P*_*t*_, where Δ*P*_*t*_ = *P*_*t*_−*P*_*t*−1_. The information trader does investment after considering how private information affects the price *P*_*t*_, instead of the price change Δ*P*_*t*_. When new information shocks the stock, the information trader enters the market with high expectations and following the “buy and hold” principle, which implies that the information trader closes out the stock till survival. As the accounting information is uncovered quarterly, we assume that the information trader is interested in long-term investment and the survival of the information trader is much longer than the survival of the sentiment trader.

(ii) The sentiment trader

The sentiment trader optimizes his or her investment by the sentiment which is affected by the price change. This type of trader also follows the “buy and hold” principle and the survival period is *k*. As the price changes more frequently than the uncovered accounting information, we assume that the survival period *k* of the sentiment trader is much shorter than the survival period of the information trader, and also much shorter than the whole period T.

Furthermore, the sentiment trader is divided into the optimistic trader and the pessimistic trader. When the stock price is on the rise, the investor who feels optimistic and enters the market is the optimistic trader. When the stock price increases Δ*P*_*t*−1_ in *t*−1 time, the optimistic trader enters the market to buy the stock, which leads the stock price to increase *W*_*o*_Δ*P*_*t*−1_ at the time *t*. Where *W*_*o*_>0, *W*_*o*_ is the elasticity coefficient that implies the level of price change caused by the optimistic trader. As the survival period of the sentiment trader is *k*, the optimistic trader who enters the market at the time *t*−1 should exit the market at the time *t*+*k*−1, which leads the stock price to decline *W*_*o*_Δ*P*_*t*−1_. On the other hand, when the stock price is falling, the investor who feels pessimistic and enters into the market to short selling stock is the pessimistic trader. When the stock price decreases Δ*P*_*t*−1_ in *t*−1 time, the pessimistic trader enters the market to sell the stock, leading the stock price to decrease *W*_*p*_Δ*P*_*t*−1_. Where *W*_*p*_>0, *W*_*p*_ is the elasticity coefficient: the level of price change caused by the pessimistic trader. As the survival period of the sentiment trader is *k*, the pessimistic trader who enters the market in *t*−1 time exit the market in *t*+*k*−1 time, leading the stock price to increase *W*_*p*_Δ*P*_*t*−1_. Furthermore, we assume *W*_*o*_+*W*_*p*_<1, which means that the effect of the sentiment trader on the stock price is less than the price change.

(iii) The noise trader

The noise trader is the investor who is the counterparty against the information trader or the sentiment trader and only provides stock quantity *Q* every time. This assumption is similar to the assumption in the HS model [33]. Considering the real financial market, the behavior of the noise trader only leads the stock price to fluctuate slightly and does not attract the sentiment trader to enter the market.

Finally, from the evidence provided by Hong and Stein [33], this paper assumes that the information spreads gradually among all information traders. The information spread process is uniformed and needs *λ* periods. The effect of information on the stock’s basic value is noted as *ε*_*t*_. According to the assumption, when the information *ε*_*t*_ appears at the time *t*, only *ε*_*t*_*/λ* parts of the information are observed at the time *t*, which leads the stock price to change *ε*_*t*_*/λ*. At the time *t*+1, other *ε*_*t*_*/λ* parts of the information are observed, which leads that the stock price changes *ε*_*t*_*/λ* as well. Until after *λ* periods, at the time *t*+*λ*, the information *ε*_*t*_ is observed completely. At the time *t*+*λ*, the effect of the information on stock price is accomplished. The overall effect of information on the stock price is *ε*_*t*_.

## 3. The asset pricing model

Denote that *S*_0_ is the initial value of the stock, *ε*_*i*_ is a piece of positive information appearing at the time *i*; *P*_*t*_ is the stock price at the time *t*; *θQ* is the effect of noise trader on stock price and *θ* is a coefficient that varies with the risk aversion of the noise trader. Under the assumptions in section 2, we know that the price of the stock at time t should be equal to the initial price combined with the effects of the three types of investors. Thus, we construct a recursive dynamic asset pricing model with the information trader, the sentiment trader and the noise trader as following: The stock price and the change of the price at the time *t* are

$$P_{t}=S_{0}+(\sum_{i=0}^{t-\lambda }\varepsilon_{i}+\sum_{j=1}^{\lambda -1}\frac{j\varepsilon_{t-j}}{\lambda })+\sum_{l=1}^{k}W\Delta P_{t-l}-\theta Q.$$ (1)

$$\Delta P_{t}=P_{t}-P_{t-1}=\frac{1}{\lambda }\sum_{j=0}^{\lambda -1}\varepsilon_{t-j}+W(\Delta P_{t-1}-\Delta P_{t-k-1}).$$ (2)

Where, in the formula (1), $\sum_{i=0}^{t-\lambda }\varepsilon_{i}+\sum_{j=1}^{\lambda -1}\frac{j\varepsilon_{t-j}}{\lambda }$ is the price change caused by the information trader from time 0 to time *t*−1, and $\sum_{l=1}^{k}W\Delta P_{t-l}$ is the price change caused by the sentiment trader from time *t*−*k* to time *t*−1. *W*>0 is the elasticity coefficient of the sentiment investor for the price change. If the sentiment investor is optimistic, then *W* = *W*_*o*_. If the investor is pessimistic, then *W* = *W*_*p*_. Obviously, $S_{0}+\sum_{i=0}^{t-\lambda }\varepsilon_{i}+\sum_{j=1}^{\lambda -1}\frac{j\varepsilon_{t-j}}{\lambda }$ is the basic value of the stock at the time *t*. And, from Eq (2), we know that $\Delta P_{t}-W\Delta P_{t-1}+W\Delta P_{t-k-1}=\frac{1}{\lambda }\sum_{j=0}^{\lambda -1}\varepsilon_{t-j}$, which shows that the stock price change is a process of ARMA(*k*+2,*λ*) and the price change Δ*P*_*t*_ is the recursive element. The appendix proves that our recursive dynamic asset pricing model with heterogeneous traders is stationary when *W*∈(0, 1).

Additionally, considering a particular case that *W*>1, and without loss of generality; assuming Δ*P*_1_>0 and no other information shocks the stock basic value (*ε*_*t*_ = 0). Then, from Eq (2), we have

$$\left\{ \begin{array}{ll} \Delta P_{t}=W^{t-1}\Delta P_{1} & t\leq k+1\\ \Delta P_{t}=(W^{t-1}-W)\Delta P_{1} & t\geq k+2 \end{array}\right.$$

Obviously, if *t*→∞ and the economic environment does not change, then we have Δ*P*_*t*_→∞ and *P*_*t*_→∞ (Because *W*>1). The market price of a stock is much higher than its true value, which implies a bubble. *P*_*t*_→∞, this particular case is treated as a serious asset bubble. If the bubble bursts, it could easily trigger a financial crisis. Because *W* is the elasticity coefficient of the sentiment investor for the stock change, we conclude that the asset bubble and financial crisis are caused by investor sentiment.

## 4. The dynamic process of price change

In this section, the process of price change under a shock of information is described. Without loss of generality, we assume that the information trader and the sentiment investor do not affect the stock price before the initial time *t*. Note that $S_{t}=S_{0}+\sum_{i=0}^{t-\lambda }\varepsilon_{i}=S_{t-1}$, then the stock price at the time *t* is

$$P_{t}=S_{t}-\theta Q$$ (3)

From Eq (3), the price change is Δ*P*_*t*_ = *P*_*t*_−*P*_*t*−1_ = 0. Assuming that a piece of positive information $\varepsilon_{t}^{\prime }$ appears at the time *t*, the spreading process of information is uniformed and needs *λ* periods. The information trader buys the stock, which leads the stock price to increase $\varepsilon_{t}^{\prime }/\lambda$ at the time *t*+1. That is

$$P_{t+1}=S_{t}+\frac{\varepsilon_{t}^{\prime }}{\lambda }-\theta Q$$ (4)

From Eqs (3) and (4), the inequality $\Delta P_{t+1}=P_{t+1}-P_{t}=\varepsilon_{t}^{\prime }/\lambda >0$ is satisfied. The optimistic trader is attracted and enters the market at the time *t*+1.

(I) Between periods [*t*+2, *t*+*λ*]

Since the information needs *λ* periods to spread among all information trader, the information trader should enter the market between [*t*+1, *t*+*λ*]. The stock price is rising steadily which keeps attracting the optimistic trader to enter the market and buy stock from the time *t*+1. Therefore, for any *m*∈[*t*+2, *t*+*λ*], we know that

$$P_{m}=S_{t}+\frac{m-t}{\lambda }\varepsilon_{t}^{\prime }+\sum_{i=t}^{m-1}W_{o}\Delta P_{i}-\theta Q,$$ (5)

and

$$\Delta P_{m}=P_{m}-P_{m-1}=\frac{\varepsilon_{t}^{\prime }}{\lambda }\frac{1-W_{0}^{m-t}}{1-W_{0}}>0.$$ (6)

The stock price should be rising steadily in the periods [*t*+2, *t*+*λ*]. Because $\Delta P_{m}-\Delta P_{m-1}=(\varepsilon_{t}^{\prime }/\lambda )W_{o}^{m-t-1}>0$, the increase in stock price is accelerating. Denote *PO*_*m*_ as the effect of the optimistic traders in time *m*, then *PO*_*m*_ is

$$PO_{m}=\Delta P_{m}-\frac{\varepsilon_{t}^{\prime }}{\lambda }=\frac{\varepsilon_{t}^{\prime }}{\lambda }\cdot \frac{W_{o}-W_{o}^{m-t}}{1-W_{o}}.$$ (7)

(II) Between periods [*t*+*λ*+1, *t*+*k*+1]

After the time *t*+*λ*, the positive information $\varepsilon_{t}^{\prime }$ has spread completely, and all information traders have entered the market, thus the basic value of the stock is $S_{t}+\varepsilon_{t}^{\prime }$ at the time *t*+*λ*. From Eq (6), we know that $\Delta P_{t+\lambda }=(\varepsilon_{t}^{\prime }/\lambda )[(1-W_{o}^{\lambda })/(1-W_{o})]>0$, so the optimistic trader is still attracted to enter the market at the time *t*+*λ*+1, which causes Δ*P*_*t*+*λ*+1_>0. Therefore, for any *n*∈[*t*+*λ*+1, *t*+*k*+1], we have

$$P_{n}=S_{t}+\varepsilon_{t}^{\prime }+\sum_{i=t}^{n-1}W_{o}\Delta P_{i}-\theta Q,$$ (8)

$$\Delta P_{n}=P_{n}-P_{n-1}=W_{o}^{n-t-\lambda }\cdot \Delta P_{t+\lambda }=W_{o}^{n-t-\lambda }\cdot \frac{\varepsilon_{t}^{\prime }}{\lambda }\cdot \frac{1-W_{0}^{\lambda }}{1-W_{0}}>0.$$ (9)

From Eq (9), the price change is greater than zero at any time between [*t*+*λ*+1, *t*+*k*+1], which implies that the optimistic trader is still attracted to enter the market to buy stock between [*t*+*λ*+1, *t*+*k*+1]. This behavior of optimistic trader leads the price to increase continuously. But $\Delta P_{n}-\Delta P_{n-1}=W_{o}^{n-t-\lambda -1}\cdot (W_{o}-1)\cdot \frac{\varepsilon_{t}^{\prime }}{\lambda }\cdot \frac{1-W_{0}^{\lambda }}{1-W_{0}}<0$, which implies that the price increases are gradually flattening out. Denote the effect of the optimistic trader at the time *n* to be *PO*_*n*_. Only the optimistic trader affects the stock price between periods [*t*+*λ*+1, *t*+*k*+1], from Eq (9), *PO*_*n*_ is

$$PO_{n}=\Delta P_{n}=W_{o}^{n-t-\lambda }\cdot \frac{\varepsilon_{t}^{\prime }}{\lambda }\cdot \frac{1-W_{0}^{\lambda }}{1-W_{0}}.$$ (10)

On the other hand, from Eqs (8) and (9), we have

$$P_{t+k+1}=S_{t}+\varepsilon_{t}^{\prime }+\sum_{i=t+1}^{t+k}W_{o}\Delta P_{i}-\theta Q,$$ (11)

$$\Delta P_{t+k+1}=W_{o}^{k-\lambda +1}\cdot \frac{\varepsilon_{t}^{\prime }}{\lambda }\cdot \frac{1-W_{o}^{\lambda }}{1-W_{o}}.$$ (12)

Because *k*<<*λ* and 0<*W*_*o*_<1, Δ*P*_*t*+*k*+1_→0 is satisfied. In other words, price changes slightly at the time *t*+*k*+1, and does not attract the optimistic trader to enter the market (In the next section, we will give an example in which the basic value of the stock is 50 and the threshold of the price change is 0.05.). However, as the sentiment investor follows the “buy and hold” principle and the survival period of the sentiment trader is *k*, the optimistic trader who enters the market at the time *t*+1 should exit the market at the time *t*+*k*+1. Because when the sentiment trader enters or exits the market, the entering or exiting behavior equally affects the stock price in opposite directions, the stock price should decline after the time *t*+*k*+2. The falling of the price attracts the pessimistic trader to enter the market to do short-selling, which leads the stock price to further decline.

(III) Between periods [*t*+*k*+2, *t*+2*k*+1]

The optimistic trader who enters the market at the time *t*+1 should exit the market at the time *t*+*k*+1, and when the sentiment trader enters or exits the market, the entering or exiting behavior equally affects the stock price in opposite directions, from Eq (11), we know that

$$P_{t+k+2}=S_{t}+\varepsilon_{t}^{\prime }+\sum_{i=t+2}^{t+k+1}W_{o}\Delta P_{i}-\theta Q,$$

$$\Delta P_{t+k+2}=-W_{o}\Delta P_{t+1}=-W_{o}\cdot \frac{\varepsilon_{t}^{\prime }}{\lambda }<0.$$

The above inequality implies that the pessimistic trader should enter the market to do short-selling. The effects on the stock price are different when the optimistic trader enters the market between periods [*t*+2, *t*+*λ*] and between periods [*t*+*λ*+1, *t*+*k*+1]. The optimistic trader exits the market, which also has an impact on stock price between periods [*t*+2, *t*+*λ*] and between periods [*t*+*λ*+1, *t*+*k*+1]. Therefore, for any time *x*, *x*∈[*t*+*k*+2, *t*+2*k*+1],

$$P_{x}=S_{t}+\varepsilon_{t}^{\prime }+\sum_{i=x-k}^{t+k+1}W_{o}\Delta P_{i}-\theta Q+\sum_{j=t+k+1}^{x-1}W_{p}\Delta P_{j},$$ (13)

$$\Delta P_{x}=-W_{o}\Delta P_{x-k-1}+W_{p}\Delta P_{x-1}=-W_{o}\sum_{i=t+1}^{x-k-1}W_{p}^{x-k-1-i}\Delta P_{i}.$$ (14)

For any *t*′∈[*t*+1, *t*+*k*], from Eq (6), Δ*P*_*t*′_>0 is satisfied. The survival of the optimistic trader is *k*, Δ*P*_*x*_<0 for any *x*∈[*t*+*k*+2, *t*+2*k*+1], which implies that the stock price should decline between periods [*t*+*k*+2, *t*+*k*+*λ*]. Furthermore, for any *x*∈[*t*+*k*+2, *t*+*k*+*λ*], from Eq (14), we know that Δ*P*_*x*_−Δ*P*_*x*−1_<0, which implies that the stock price accelerates to fall. On the other hand, from Eq (14), we know that Δ*P*_*t*+2*k*+1_≈0, which implies that the change in the stock price is slight at the time *t*+2*k*+1 and does not attract the pessimistic trader to enter the market.

(IV) Between periods [*t*+2*k*+3, *t*+3*k*+1]

As aforementioned, Δ*P*_*t*+2*k*+1_≈0, we know that the pessimistic trader should not enter the market at the time *t*+2*k*+1. However, the sentiment trader follows the “buy and hold” principle and the survival period is *k*, so the pessimistic trader who enters the market at the time *t*+*k*+2 should exit the market by buying the stock at *t*+2*k*+2. Thus, the behavior of the pessimistic trader leads the stock price to increase and the increasing level is equal to −*W*_*p*_Δ*P*_*t*+*k*+2_ (the level that the pessimistic trader enters the market at the time *t*+*k*+2). In other words, the price change caused by the pessimistic trader at the time *t*+*k*+3 is Δ*P*_*t*+2*k*+3_ = −*W*_*p*_Δ*P*_*t*+*k*+2_>0. Then the increasing stock price attracts the optimistic trader to enter the market by buying the stock at the time *t*+2*k*+3. This is a new fluctuation of the stock price, noted as the second fluctuations. At the beginning of the second fluctuation, the pessimistic trader who enters the market in the first fluctuation and the optimistic trader who is attracted by the increasing stock price in the second fluctuation should cause the increase of stock price. For any *y*∈[*t*+2*k*+3, *t*+3*k*+1],

$$P_{y}=S_{t}+\varepsilon_{t}^{\prime }-\theta Q+\sum_{i=y-k}^{t+2k}W_{p}\Delta P_{i}+\sum_{j=t+2k+2}^{y-1}W_{o}\Delta P_{j},$$ (15)

$$\Delta P_{y}=-W_{p}\Delta P_{y-k-1}+W_{o}\Delta P_{y-1}.$$ (16)

From Eq (15), obviously, $\Delta P_{y}=-W_{p}\sum_{j=t+k+2}^{y-k-1}W_{o}^{y-k-1-j}\Delta P_{j}>0$ is satisfied. Thus, the pessimistic trader exits the market by buying the stock and the optimistic trader enters the market also by buying the stock between periods [*t*+2*k*+3, *t*+3*k*+1], which leads the stock price to increase. Furthermore, from Eqs (15) and (16), we have

$$P_{t+3k+1}=S_{t}+\varepsilon_{t}^{\prime }-\theta Q+\sum_{j=t+2k+3}^{t+3k}W_{o}\Delta P_{j},$$

$$\Delta P_{t+3k+1}=-W_{p}(\Delta P_{t+2k+1}+W_{o}\Delta P_{t+2k+2}+\cdots +W_{o}^{k-1}\Delta P_{t+k+2})\approx 0.$$

The above formula implies that the stock price changes slightly at the time *t*+3*k*+1, which does not attract the sentiment investor to enter the market. The survival period of the sentiment investor is *k*, so the optimistic trader who enters the market at the time *t*+2*k*+3 should exit the market by selling the stock at the time *t*+3*k*+3. This behavior makes the stock price decline at the time *t*+3*k*+4. Thus, the pessimistic trader enters the market by short-selling and leads the stock price to decline further. This is the declining wave of the second fluctuation. When the optimistic trader exits the market in the second fluctuation, the stock price changes slightly and does not attract the pessimistic trader to enter the market. After *k* periods, the pessimistic trader who enters the market by short-selling in the second fluctuation should exit the market by buying the stock, which leads the stock price to increase again and attracts the optimistic trader to enter the market by buying the stock again, etc. In other words, the sentiment investor enters and exits the market repeatedly, which causes the stock price to fluctuate. The repeated movement of sentiment investor in and out of the market is the main reason for the sustainability of stock price fluctuations.

(V) A summary of the stock price fluctuation

As discussed above, positive information shocks the stock price to fluctuate strongly and continuously under the effect of the sentiment investor. To understand the fluctuation process, a summary is presented as follows:

- Between periods [*t*+1, *t*+*k*+1]. Under a shock of positive information, the information trader and the optimistic trader buy the stock, accelerating the increase of stock price.
- Between periods [*t*+*k*+2, *t*+2*k*+1]. The optimistic trader exits the market by selling the stock and the pessimistic trader enters the market by short-selling, causing the stock price to continue to decline. Furthermore, the stock price accelerates to fall between periods [*t*+*k*+3, *t*+*k*+*λ*].
- Between periods [*t*+2*k*+3, *t*+3*k*+1]. The pessimistic trader exits the market by short-selling and the optimistic trader enters the market by short-selling, causing the stock price to increase continuously. Furthermore, the stock price accelerates to increase between periods [*t*+2*k*+4, *t*+2*k*+*λ*].

Finally, the behavior of the sentiment investor who enters or exits the market is the main factor that leads the stock price to fluctuate powerfully and continuously, causing the sustainability of stock price fluctuations anomaly. The result shows that the price fluctuation process of the model is in line with the real financial market.

On the other hand, if there is no sentiment trader in the market, then the stock price *P*_*t*′_ is

$$P_{t^{\prime }}=S_{t}+\frac{t^{\prime }-t}{\lambda }\varepsilon_{t}^{\prime }-\theta Q,\;t'\leq t+\lambda$$ (17)

$$P_{t'}=S_{t}+\varepsilon_{t}^{\prime }-\theta Q.\;t'\geq t+\lambda$$ (18)

Comparing Eqs (17) and (18) with the stock price Eq (1) which is affected by the sentiment investor, we find that if there is no sentiment trader in the market, the stock price fluctuates slightly under the shock of information. This fluctuation with no sentiment influence is consistent with the standard finance theory. But if there is a sentiment trader in the market, the stock price fluctuates strongly and causes the sustainability of stock price fluctuations anomaly. Therefore, unlike other classical theoretical asset pricing models based on the heterogeneous [23–30], we conclude that the sustainability of stock price fluctuations is caused by the sentiment investor, which is consistent with the empirical results of [17–22].

Meanwhile, from Eq (18), we know that the basic value (*P*_*B*_) of the asset is $S_{t}+\varepsilon_{t}^{\prime }$, the highest price (*P*_*H*1_) of the stock is $S_{t}+\varepsilon_{t}^{\prime }-\theta Q$ when there is no sentiment trader in the market. And the highest price (*P*_*H*2_) of the stock is $S_{t}+\varepsilon_{t}^{\prime }+\sum_{i=t}^{n-1}W_{o}\Delta P_{i}-\theta Q$ when the price is affected by investor sentiment. The shock is caused by positive information and *W*_*o*_>0 is assumed, thus *P*_*H*2_>*P*_*H*1_ is satisfied. In other words, the overreaction of stock price to a piece of good news is caused by the sentiment investor, which is consistent with the seminal work of Barberis, et al. [34] and the recent empirical results (Piccoli and Chaudhury [35]; Seok, et al. [36] and Parveen, et al. [37]). An asset bubble can be described as the price of an asset substantially exceeds its basic value. Comparing *P*_*B*_ with *P*_*H*2_, we find that only if *W*_*o*_ is great enough, that *P*_H2_>*P*_*B*_ the price of an asset substantially exceeds its basic value. Therefore, we conclude that the asset bubble is due to investor sentiment which is consistent with Pan [38] who confirms that investor sentiment positively reacts to bubble shocks. Furthermore, it is noticeable that a shock from negative news should also lead the stock price to fluctuate and should also cause the sustainability of stock price fluctuations and overreaction.

## 5. Examples

To describe the fluctuation of the stock price and explain the sustainability of stock price fluctuations and overreaction, an example is provided in this section.

(i) Numerical Simulation of the Model

In the numerical simulation, we assume that a piece of positive information shocks the stock price, and the stock price fluctuation is influenced by the heterogeneous traders (the information trader, the sentiment trader and the noise trader) as discussed in the theoretical model. As the information is positive, the optimistic trader has a greater elasticity coefficient for the stock price change than the pessimistic trader. Specifically, the parameters used in the numerical simulation are shown in Table 1.

**Table 1:** The parameter direction of the numerical simulation.

Symbols	Implications	Valuations
*t*	The time that positive information appears.	1
$\varepsilon_{t}^{\prime }$	The effect of positive information on the stock price.	5
*λ*	The spreading periods of the information.	3
*P*_1_	The stock price at the time 1.	50
*k*	The survival period of the sentiment trader.	50
*W*_*o*_	The elasticity coefficient of optimistic traders.	0.5, 0.6
*W*_*p*_	The elasticity coefficient of the pessimistic traders.	0.2, 0.3

Note: Because *W*_*o*_+*W*_*p*_<1, *W*_*o*_,*W*_*p*_∈(0, 1) and a piece of positive information shocks the stock price, we choose *W*_*o*_>*W*_*p*_. The threshold of the price change is 0.05, which means that if the price change less than 0.05, the sentiment trader is not attracted to enter the market.

**Fig 2:**
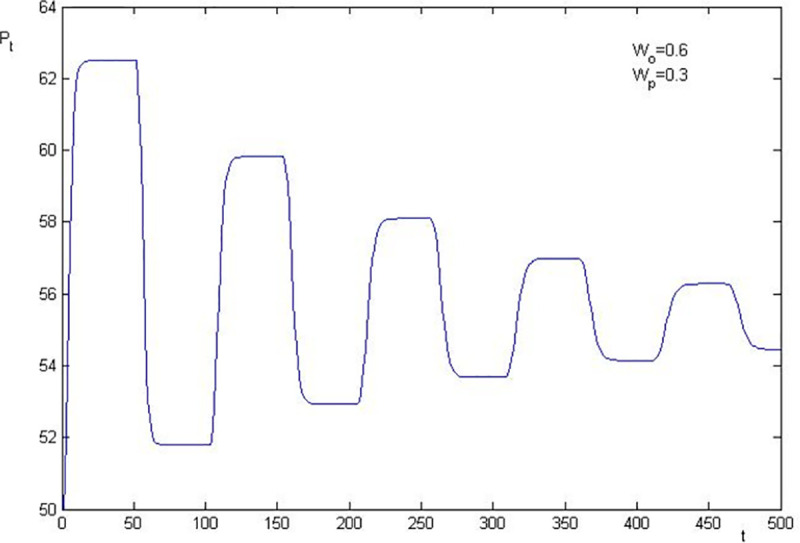
Price fluctuation when W0 = 0.6, Wp = 0.3.

**Fig 3:**
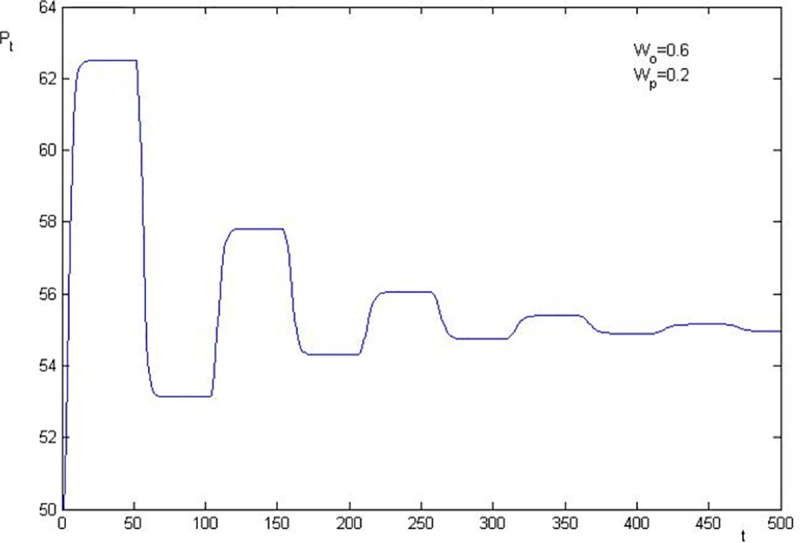
Price fluctuation when W_0_ = 0.6, W_p_ = 0.2.

**Fig 4:**
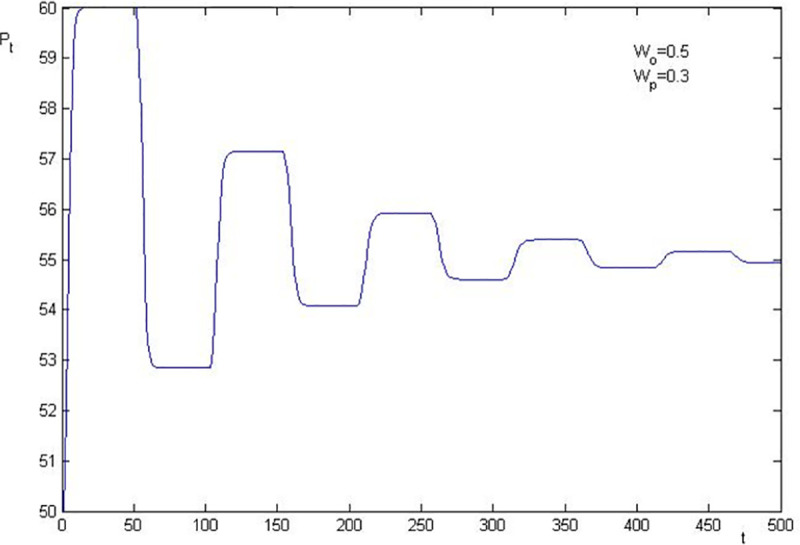
Price fluctuation when W_0_ = 0.5, W_p_ = 0.3.

**Fig 5:**
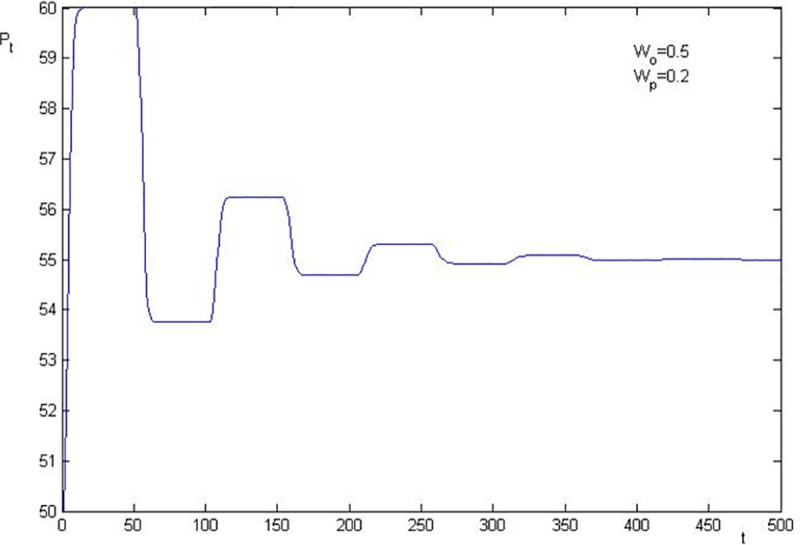
Price fluctuation when W_0_ = 0.5, W_p_ = 0.2.

Using the parameters in Table 1, we know that the basic value of the stock price after the shock of information is 55 in the simulation. The numerical simulations of stock price fluctuation are presented in Figs 2–5. The results are as follows:

The level that stock price deviates from the basic value is closely related to the elasticity coefficient of the sentiment trader on stock price change. The larger the elasticity coefficients are, the higher the level of deviation is. In Fig 3, *W*_0_ = 0.6 and *W*_*p*_ = 0.2, the highest stock price is 62.5 (1.14 times over the basic value), and the lowest stock price is 51.79 (0.94 times of the basic value). Thus, we conclude that the asset bubble, overreaction and financial crisis are attributable to investor sentiment, since the stock price deviates from the basic value which is caused by sentiment investor.

The stock price convergence and the elasticity coefficient of the sentiment trader are negatively correlated. By comparing Fig 2 with Fig 3, and Fig 4 with Fig 5, we find that the larger the elasticity coefficient of the sentiment trader is, the slower the stock price convergence is. In particular, in Fig 2, where *W*_0_ = 0.6 and *W*_*p*_ = 0.3, after 500 periods, the stock price is still fluctuating. Moreover, a particular simulation shows that the stock price converges after 1045 periods which is 21 times as much as the survival period of the sentiment trader. However, in Fig 5, *W*_0_ = 0.5 and *W*_*p*_ = 0.2, the stock price converges after 368 periods which is only 7 times as much as the survival period of the sentiment trader. All these imply that investor sentiment is the key factor that leads the stock price to fluctuate powerfully and causes the sustainability of stock price fluctuations anomaly.

To sum up, the numerical simulation also indicates that investor sentiment is the key factor that causes the stock price to fluctuate. The effect level of the sentiment is positively correlated with the fluctuation level of the stock price, which leads to the sustainability of stock price fluctuations. Furthermore, we show that overreaction, asset bubble and the financial crisis are also attributable to investor sentiment.

(ii) Robustness

The above subsection shows that investor sentiment is the key factor that causes price fluctuation. Other parameters that may affect the paper’s conclusions are: One is $\varepsilon_{t}^{\prime }$, the effect of the positive information on the stock price. Another parameter is *λ*, the spreading time of the information. These two parameters are related to “information”, and their value may affect the stock price change in the initial stage of fluctuation. The last parameter is *k*, the survival period of the sentiment trader. The value of the parameter *k* directly affects the fluctuation of the stock price. Do the changes in these three parameters affect the results? The robustness of the results is tested by changes in these parameters in Table 2.

**Table 2:** The effects of the parameters on the stock price.

Panel A:	*λ* = 3, *k* = 50, *W*_0_ = 0.6, *W*_*p*_ = 0.3, *t* = 1, *P*_1_ = 50					
$\varepsilon_{t}^{\prime }$	Intrinsic value	Highest price	Lowest price	Away from the basic value	Fluctuating periods	Convergence
5	55	62.50	51.79	19.48%	1045	21
7	57	67.50	52.50	26.32%	1096	22
9	59	72.50	53.21	32.69%	1146	22
Panel B:	$\varepsilon_{t}^{\prime }=5$, *k* = 50, *W*_0_ = 0.6, *W*_*p*_ = 0.3, *t* = 1, *P*_1_ = 50					
*λ*	Basic value	Highest price	Lowest price	Away from the basic value	Fluctuating periods	Convergence
2	55	62.50	51.79	19.48%	1093	22
4	55	62.50	51.79	19.48%	1094	22
6	55	62.50	51.79	19.48%	1044	21
Panel C:	$\varepsilon_{t}^{\prime }=5$, *λ* = 3, *W*_0_ = 0.6, *W*_*p*_ = 0.3, *t* = 1, *P*_1_ = 50					
*k*	Basic value	Highest price	Lowest price	Deviation of basic value	Fluctuating periods	Convergence
40	55	62.50	51.79	19.48%	797	20
60	55	62.50	51.79	19.48%	1306	22
80	55	62.50	51.79	19.48%	1727	22

Note: In the table, “Deviation of basic value” = (Highest price- Lowest price)/Basic value, and “Convergence” = Fluctuating periods/the survival period of the sentiment trader. From Panel A to Panel C, one parameter ($\varepsilon_{t}^{\prime }$, *λ* or *k*) is changed to simulate the stock price fluctuation.

From Table 2, we find that *λ* (the spreading term of the information) and *k* (the survival term of the sentiment trader) don’t significantly impact the fluctuation level of the stock price and the duration of the price fluctuation. And $\varepsilon_{t}^{\prime }$ (the effect of the positive information on the stock price) only affects the basic value of the stock rather than the fluctuation level of the stock price and the duration of the price fluctuation. In conclusion, the results are robust. The behavior of the sentiment investor who enters or exits the market is the main factor that leads the stock price to fluctuate wildly and continuously and causes the sustainability of stock price fluctuations [22], the overreaction [35–37], the asset bubble [38], even the financial crisis.

## 6. Conclusions

This paper derives a recursive dynamic asset pricing model with heterogeneous traders: the information trader, the sentiment trader and the noise trader. A theoretical discussion and a numerical simulation for the fluctuation of the stock price under the shock of information are given. The paper finds that investor sentiment is the key factor that leads the stock price to fluctuate powerfully and causes the sustainability of stock price fluctuations anomaly. The level that the stock price deflects the basic value is closely related to the elasticity coefficient of the sentiment trader on the stock price change. The greater the elasticity coefficient is, the higher the level that the stock price deflects the basic value is. Furthermore, the stock price convergence is closely related to the elasticity coefficient of the sentiment trader on the stock price change. The greater the elasticity coefficient of the sentiment trader is, the slower the stock price converges.

In brief, unlike other classical theoretical asset pricing models based on the heterogeneous [23–30], this paper depicts the fluctuation process of stock price under the common-effect of the information trader, the sentiment trader and the noise trader, proving that the investor sentiment is the key factor that causes the sustainability of stock price fluctuations anomaly [22]. Meanwhile, the asset price affected by investor sentiment is always overreacting [35–37]. However, the paper restricts the value of the elasticity coefficients of the sentiment trader on the stock price change. If these restrictions are elevated, the stock price may no longer converge or the convergence time is too long, which could explain the asset bubble [38] and the financial crisis caused by investor sentiment. This paper adopts the recursive method to build a dynamic asset pricing model based on heterogeneous investors. Compared with seminal works [23–30], this model is easier to simulate the trend of asset prices after information shock. It can intuitively show the persistence of asset price fluctuation through numerical simulation, and also shows that the sustainability of stock price fluctuations, the overreaction, the asset bubble, even the financial crisis are mainly caused by sentiment investor. This paper provides a new perspective for academics to construct dynamic asset pricing model based on heterogeneous, and provides theoretical basis for professionals in the financial industry to construct their quantitative investment strategy, and they should all pay more attention to investor sentiment, which is an important factor in the asset pricing.

However, this paper only discusses a recursive dynamic asset pricing model with three types of investors traders (the information trader, the sentiment trader and the noise trader). For the further direction of research, one is to further study the impact of other types of investors on stock price by using recursive method. The second is combining with the latest data of the Covid-19’s impact on stock price to empirically test the conclusion of the paper.

## Supporting information

S1 File Program for the numerical simulation. (RAR)

S1 Appendix (DOCX)
