# Supplementary material for: The sustainability of stock price fluctuations: Explanation from a recursive dynamic model
Source: PLoS One. 2021 Aug 17;16(8):e0255081. doi: 10.1371/journal.pone.0255081 (PMC8370623; doi:10.1371/journal.pone.0255081)
Supplement: S1 Appendix — (DOCX) [file pone.0255081.s002.docx]

# Appendix

The proof of that the Eq. is stationary.

Because Eq. is equivalent to Eq., the stationary condition of the Eq. follows that the roots $x$ of

$1-Wx+Wx^{k+1}=0$ A(1)

must lie outside the unit circle (e.g., , Hamilton [39], Hong and Stein [32]). In other words, we only need to prove that there are no roots $x$ of Eq. A(1) in the unit circle. Because $0<W<1$, we know that:

(1) If $0\leq x\leq1$, then $1-W(x-x^{k+1})>1-W>0$.

(2) If $-1\leq x<0$, then $1-Wx+Wx^{k+1}>1+Wx^{k+1}>0$.

Hence, Eq. is stationary.
